# The Reasons behind the (Non)Use of Feedback Reports for Quality Improvement in Physical Therapy: A Mixed-Method Study

**DOI:** 10.1371/journal.pone.0161056

**Published:** 2016-08-12

**Authors:** Marijn Scholte, Catherina W. M. Neeleman-van der Steen, Philip J. van der Wees, Maria W. G. Nijhuis-van der Sanden, Jozé Braspenning

**Affiliations:** 1 Scientific Institute for Quality of Healthcare Radboud Institute for Health Sciences, Radboud University Medical Center, Nijmegen, The Netherlands; 2 ROS Caransscoop, Beekbergen, The Netherlands; University of Liverpool, UNITED KINGDOM

## Abstract

**Objectives:**

To explain the use of feedback reports for quality improvements by the reasons to participate in quality measuring projects and to identify barriers and facilitators.

**Design:**

Mixed methods design.

**Methods:**

In 2009–2011 a national audit and feedback system for physical therapy (Qualiphy) was initiated in the Netherlands. After each data collection round, an evaluation survey was held amongst its participants. The evaluation survey data was used to explain the use of feedback reports by studying the reasons to participate with Qualiphy with correlation measures and logistic regression. Semi-structured interviews with PTs served to seek confirmation and disentangle barriers and facilitators.

**Results:**

Analysis of 257 surveys (response rate: 42.8%) showed that therapists with only financial reasons were less likely to use feedback reports (OR = 0.24;95%CI = 0.11–0.52) compared to therapists with a mixture of reasons. PTs in 2009 and 2010 were more likely to use the feedback reports for quality improvement than PTs in 2011 (OR = 2.41;95%CI = 1.25–4.64 respectively OR = 3.28;95%CI = 1.51–7.10). Changing circumstances in 2011, i.e. using EHRs and financial incentives, had a negative effect on the use of feedback reports (OR = 0.40, 95%CI = 0.20–0.78). Interviews with 12 physical therapists showed that feedback reports could serve as a tool to support and structure quality improvement plans. Barriers were distrust and perceived self-reporting bias on indicator scores.

**Conclusions:**

Implementing financial incentives that are not well-specified and well-targeted can have an adverse effect on using feedback reports to improve quality of care. Distrust is a major barrier to implementing quality systems.

## Introduction

Measuring the quality of care with quality indicators (QIs) has become part of the care giving process nowadays [1]. The QIs presented in a feedback report in which scores can be compared to a benchmark are proposed to be a driving force to improve the quality of care with best practices as a guide [2–8]. Until recently, studies mainly focused on the question whether feedback reports influenced quality improvements, rather than why. Studying success factors of quality improvement initiatives in different healthcare settings, Kaplan [9] showed that micro-system motivation, i.e. the willingness and desire of health care professionals to improve performance, had a major influence in predicting success. A qualitative study into the barriers and facilitators to use feedback reports for quality improvements [10] indicated that fostering loyalty and retaining patients was a more important drive to improve the quality of care than public reporting and the use of feedback reports. External incentives such as pay-for-performance incentives were found to be important motivators for the use of feedback reports to improve the quality of care [10]. However, several reviews of studies into the effects of financial stimuli in health care provide little evidence that financial rewards have effect on the behavior of physicians and furthermore, the evidence that was found could not be generalized due to weak research designs [11–13]. Other research suggests however that extrinsic stimulation such as financial stimuli can also decrease intrinsic motivation, that is motivation that comes from within the person, especially when the initial intrinsic motivation is high [14,15]. It can even have an adverse effect on the desired behavior, as feelings of self-control suffer from the influence of external sources trying to control the behavior [14,15]. This seems particularly the case when incentives are not well-specified or well-targeted [16]. Research into the effect of financial incentives on health related behavior does not show such an adverse effect, it can rather serve as a reward for the individual’s autonomous decision as opposed to a controlling mechanism for his or her behavior [17]. Intrinsic motivation is claimed to have a more natural tendency, as humans are curious, and willing to learn and explore without external incentives [14]. However, in the practice of quality improving initiatives, a combination of implementation strategies for quality improvements focusing on arousing motivating the participants from a professional perspective through education, feedback and reminders as well as through extrinsic incentives, such as financial interventions are usually employed [9]. As the disclosure of health quality data increases, the question of what triggers quality improvement best becomes noteworthy.

A unique opportunity to study the effects of motivation on desired behavior arose in the quality indicator project for physical therapist (PTs) in primary care (Qualiphy). A set of 23 QIs was developed in 2008 through consensus between PTs, patient organizations, health insurers and the inspectorate in three Delphi rounds [18]. (see Box 1). The set of QIs was implemented step-by-step nationwide in three waves from 2009 to 2011 and participants received feedback reports after each wave which included a national benchmark. These feedback reports can be a powerful incentive to improve the quality of care [19] and using these reports are a proxy for real quality improvements. After participation and receiving the feedback reports, an evaluation survey was held among a random group of participating physical therapists after each wave of data collection for Qualiphy. In that survey, respondents were asked to evaluate different parts of that particular wave of the Qualiphy project, but also to retrospectively indicate what the reasons were to participate in the first place in that wave of data collection for Qualiphy and whether or not the feedback reports were used to improve the quality of care. In 2009 participation was voluntary and mainly directed to gain insight into the level of quality, but in 2010 some healthcare insurers began rewarding PTs for their participation, and in 2011 it was a precondition for additional payment by health insurers. However, these incentives were aimed at participating in the data collection, rather than at rewarding high quality, or quality improvements. Further, there was no univocal system of rewarding and punishing practices that did not comply, as different insurers handled different systems. We hypothesized that these changing circumstances, i.e. increasing external stimuli affected the reasons for PTs to participate in Qualiphy.

**Box 1**

For the Qualiphy project (Quality Indicators for Physical therapy in primary care) 23 quality indicators were developed in consensus meetings among the Royal Dutch Society for Physical Therapy (KNGF), the national agent of Health Insurance Companies, the Dutch Patient and Consumer Federation, and the Healthcare Inspectorate. The set described quality of care in three domains: (1) physical therapy care process, (2) practice management, and (3) patient experiences. Qualiphy collected data on the quality indicators in 2009, 2010 and 2011 and was set up as a national audit and feedback system in physical therapy. In the first two years the physical therapy care process was evaluated by self-rating. In 2011 these data were extracted directly from the Electronic Health Records (EHRs). The validity and reliability of the first domain was assessed by Scholte et al. [20] for 2009 and 2010. The main issue of the indicators was the ceiling effects of all indicators. This made the distinction between high and low scoring therapists and practices more difficult.

Research on the reasons behind success or failure of quality improvement initiatives based on quality indicators is scarce and evidence for effective use of financial incentives is weak [11–13]. The aim of this study was to assess the influence of the initial motivation to participate in the Qualiphy project on the use of feedback reports for quality improvement in physical therapy. We established the following research questions:

1. What reasons do PTs have to participate in Qualiphy and do these reasons vary when financial incentives were implemented more intensively by insurers?
2. Are the feedback reports on quality indicators used by PTs as a tool to improve quality of care, and is this explained by the reasons to participate?
3. What are the perceived barriers and facilitators of PTs for using feedback reports?

As the emphasis of Qualiphy shifted more towards financial stimuli from 2009 to 2011, we expected that the proportion of PTs only mentioning external incentives as a reason to participate in Qualiphy would increase from the first cohort in 2009, to 2010 and 2011 (*hypothesis 1*). Further, following the study by Ryan and Deci [14] on the possible negative effect of financial incentives, we expected that PTs who are solely participating in order to be eligible for the financial stimuli will be less likely to use the feedback reports for quality improvement than PTs who are participating either for a mixture of reasons, or for reasons other than financial benefits (*hypothesis 2*). As a result of the expected increase in PTs participating to be eligible for financial incentives and the possible negative impact of such stimuli when misdirected and poorly specified [16], we expected that the feedback reports were less likely to be used in 2011 for quality improvements than they were in 2009 (*hypothesis 3*). A mixed methods design, including questionnaires and interviews, was used to answer these research questions and test these hypotheses.

## Methods

### Design

The mixed methods study design relies on the “collection and analysis of quantitative and qualitative data, beginning with quantitative data, giving equal weight to both types of data” [21]. For the first two research questions on the use of the feedback reports, quantitative data were used from the evaluation survey, as well as qualitative data collected in semi-structured interviews with participating PTs. Both types of data were used to answer the questions by comparing the results to see if the same conclusions are reached (triangulation) [21]. For the third research question to assess barriers and facilitators for using the quality indicators and the feedback reports, this study relies solely on qualitative data to evaluate the process (e.g., why or why not the quality indicators and feedback reports were used), thus deepening the understanding of the results of the first two research questions [21].

### Setting, recruitment and sample size

After each annual wave of data collection for Qualiphy for the calculation of the quality indicators the feedback reports were distributed. After each of those waves, and after the feedback reports were distributed, a random sample of 200 participating PTs from different outpatient physical therapy practices were invited by email to participate in a web-based evaluation survey in which the respondents were made aware of the scientific purpose of the evaluation survey. The respondents were asked retrospectively what the reasons were to participate in the Qualiphy project. Further, it was asked whether or not the respondent intended to use the feedback reports they had received. The three separate data sets were then combined into a pooled data set. Of the 600 physical therapists that were invited in total, 257 completed the whole evaluation survey (42.8%).

With respect to the qualitative data, we invited participating physical therapists by email to participate in a face to face interview. First responders were called to set up appointments. As it turned out to be difficult to make face to face appointments due to the busy schedule of the physical therapists, it was decided by the researchers to conduct the interviews by telephone. The semi-structured interviews were conducted using a topic list for reference (see Table 1) and were held in 2011 with physical therapists who participated in one or more data collection rounds of the Qualiphy project. The interviews were conducted by KN and CB and took approximately 75 minutes each. After three interviews, questions and answers were discussed by the researchers and if necessary questions were reformulated or added. It was decided to apply interviews until saturation of information was reached. The balance between positive comments and negative comments on the project as a whole, on the quality indicators and on the feedback reports was of major importance in this decision. Saturation was reached after twelve interviews as no new information was obtained during the last two interviews. Five men and seven women were interviewed with an average age of 43.8 years and an average working experience of 14.5 years. Nine PTs participated in all three years of Qualiphy, two participated in 2010 and 2011, and one participated in 2009 and 2011. Quotes have been edited for clarity and to protect respondent confidentiality. Physical therapists that participated were explained at the start of each interview that the information would be used for scientific purposes.

**Table 1:** Topic list and example questions of structured interview.

Goals for using Qis
**Experiences with Qis**
Example questions to explore facilitators and barriers
- Interested in QI feedback report?
- Why (not)?
- What do you know yet of the feedback reporting?
- What info did you read?
- Meets your expectations? Why (not)?
- Do you use the feedback for quality improvement plans? Why (not)? Which info?
- Is QI part of your quality policy?
- How did you anchor?
**Future findings for QIs and quality of PT care in general**
- Requirements
- Points for improvement
- 5- year expectance
- Idealisms

The Medical Ethical Committee Arnhem and Nijmegen waived approval for this study, as patient information was anonymous and respondents (physical therapists) gave their silent consent by participating. The study was conducted in accordance of the Declaration of Helsinki.

### Data analysis: quantitative data

The evaluation surveys consisted of between 40 and 50 questions regarding all aspects of participating in the project and data collection for the three domains of which 5 questions were about the feedback reports. Four questions of the survey were selected to answer our research questions. The answer categories differed somewhat from 2009 to 2011 (see Table 2). Our dependent variable the *use of the feedback reports as an improvement tool* was measured by the question: “Which of the following do you intend to address (first) based on the feedback reports?” The answer categories as mentioned in Table 2, were dichotomized (yes, I do intend to use the feedback report; or no, I do not intend to use the feedback reports) to assess the use of feedback reports in relation to the reasons to participate.

**Table 2:** Descriptive statistics on all relevant variables from surveys in 2009, 2010, 2011 and in total.

	2009	2010	2011	Total*
Total participating therapists in Qualiphy project (N)	7,562	6,000	9,719	11,274
Invited for evaluation survey	200	200	200	600
n (response %)	97 (48.5)	67 (33.5)	93 (46.5)	257 (42.8)
Age (Mean (standard deviation))	42.6(11.0)	43.6(11.1)	46.7(12.2)	44.3 (11.6)
Male (%)	54.6	46.3	46.2	49.4
Reasons to participate^1^				
-Improving patient care	40.2	32.8	32.3	35.4
-Increasing evidence-based practice	32.0	28.4	22.6	27.6
-Gaining insight in quality of care	57.7	52.2	45.2	51.8
-Giving structure to change in practice^2^	21.6	20.9	n.a.	21.2
-Needs from within the professional group	22.7	25.4	35.5	28.0
-Transparency	55.7	32.8	36.6	42.8
-Improving market and negotiation position	27.8	19.4	29.0	26.1
-Financial benefits	38.1	38.8	58.1	45.5
-Participation was a condition for obtaining a contract^2^	51.5	52.2	n.a.	51.7
-For the researchers	10.3	3.0	16.1	10.5
Only professional reasons (mean(sd))	0.39(0.28)	0.32(0.30)	0.28(0.27)	0.33(0.28)
Only financial reasons (mean(sd))	0.06(0.04)	0.18(0.39)	0.15(0.36)	0.13(0.33)
Mixture of reasons (mean(sd))	0.73(0.45)	0.61(0.49)	0.79(0.41)	0.72(0.45)
Action based on feedback report regarding… (%)^3^				
-Window information	11.6	7.0	15.1	10.9
-The use of measuring instruments^2^	62.8	45.6	n.a.	55.9
-Administration of methodical acting^2^^,^^4^	n.a.	21.1	n.a.	21.1
-The physical therapy process in general^4^^,^^5^	n.a.	n.a.	26.9	26.9
-Practice organization^4^^,^^5^	n.a.	n.a.	18.3	18.3
-Patient experiences^4^^,^^5^	n.a.	n.a.	29.0	29.0
-No action planned	25.6	26.3	44.1	33.1

*As there is overlap between the three years in participating physical therapists, the total is the unique number of physical therapists participating.

^1^ Multiple answers possible;

^2^Not asked in 2011;

^3^Multiple answers possible in 2011;

^4^Not asked in 2009;

^5^Not asked in 2010.

The *reasons for participating* were measured by the retrospective question “What was/were the reason(s) to participate in the Qualiphy project?” The pre-determined answer categories are listed in Table 2. To find out if there were unobserved, or latent variables underlying the reasons to participate, bivariate correlations were computed first. Six of the reasons showed significant correlations in the pooled dataset, that is all three years combined (p<0.01), as well as when the correlations were calculated for each separate years (p<0.05). Those six reasons were: *improving patient care*, *increasing evidence-based practice*, *gaining insight in quality of care*, *giving structure to change in practice*, *transparency* and *improving market and negotiation position*. Next, confirmatory factor analysis was used in SPSS Amos, using asymptotically distribution free (ADF) estimation suitable for dichotomous variables to examine whether these six items measured a latent construct. All estimates were significant (p<0.001) and model fit indexes showed a good fit (Chi-square = 8,071, df = 9, p = 0.527; TLI = 1.016). The construct was named *Professional reasons*, as they all seem to be professionally motivated reasons to participate, as opposed to financial incentives. When combined into a scale reliability analysis produced a Cronbach’s alpha of 0.671. However, as we are not interested in the influence of the degree of professional reasons, but rather what type of reasons influenced the use of feedback reports, we have constructed a dummy variable *professional reasons* for which the respondent scores 1 if he/she mentioned one of the six items of that construct and none of the others. The mean score of *only professional reasons* in the combined data set was 0.15 with a minimum score of 0 and a maximum score of 1 (See Table 2). To estimate whether *financial incentives* had an influence on the use of feedback reports for quality improvements two items were used, i.e. *financial benefits* and *participation was a condition for obtaining a contract*. A dummy variable *Only financial reasons* was calculated from these two items as a dichotomous, so that respondents that only mentioned one of these two reasons to participate would be scored 1, and respondents that either named other reasons as well, or only mentioned other reasons would be scored 0. Last, a dummy variable *mixture of reasons* was constructed for the respondents that were left. The mean score of *only financial reasons* was 0.13, meaning that 13% of all participants only had financial reasons to participate and the vast majority had a mixture of reasons, with a mean score of 0.72.

Last, year of survey and age and gender of the participants were used as control variables, as they could potentially influence the reasons to participate as well as the willingness to act on feedback.

The pooled data set of the quantitative surveys were analyzed using SPSS version 20. Chi-squares were calculated to estimate the associations between the different *reasons to participate* and *year of survey* (hypothesis 1), and between the *year of survey* and the *use of feedback reports* (hypothesis 3). For the association between the *reasons to participate* and the *use of the feedback reports* (hypothesis 2), Phi correlations were calculated. Phi correlations are directional association measures suitable for when both variables are dichotomous.

Finally, to determine whether bivariate associations would remain statistically significant while controlling for other determinants, we used logistic regression analysis using the enter method with *use of feedback reports* as the dependent variable. The first regression model only contains the dummy variables only *professional reasons* and *only financial reasons* as independent variables. A mixture of reasons to participate served as the reference category. In the second model, to do justice to the fact that data was collected in three separate years, *year of survey* was added to control for the influence of changing circumstances surrounding the Qualiphy project on the use of the feedback reports and to assess whether the effect of the reasons to participate on the use of the feedback reports would hold. Although we have data from three different years, this is not enough to use longitudinal analysis. It is recommended to use dummy variables in that case to account for the time [22]. In the third model *age* and *gender* were added as covariates. Odds ratios, 95% confidence intervals, Wald statistics and statistical significance are presented with respect to the determinants. As a last step, to determine model fit, a model Chi-square test was used comparing the full model with the constant only model. Also, to test whether each model has a better fit to the data than the previous model, the step Chi-square was calculated. Statistical significance was determined with a *p*-value < 0.05.

### Data analysis: qualitative data

All interviews were audio taped and transcribed verbatim by LB, CB and KN using F4 (v4.2 for Windows) [23]. Interviews were then summarized and sent back to the PTs for member check and possible corrections and additions. Transcripts of the interviews were openly coded and analyzed by LB and KN using Atlas.ti 6.2. The framework approach was used to analyze the data [24]. Five consecutive stages were used, the first being familiarization. In this stage, the researchers listened to all the audiotapes and read all the transcripts and notes in order to list reoccurring themes and key ideas. Second, a thematic framework was identified with all concepts, themes and key issues by which the data could be examined and referenced. The framework was discussed and finalized. In the third stage–indexing–the framework was systematically applied to all data in textual form. To increase validity, after every two, interviews were coded according to the framework by LB and KN, compared and discussed. If no consensus was reached, a third researcher (JB) had the final decision. Fourth, the data was rearranged so that the data matched the part of the framework they related to in order to form charts (charting stage). In the fifth stage, mapping and interpretation, the charts were used to define concepts, to assess the range and nature of the found phenomena and to find associations between the themes [24].

## Results

### Quantitative data

For the quantitative data, the response rate was 48.5% in 2009, 33.5% in 2010 and 46.5% in 2011 (see Table 2). Overall response rate was 42.8%. From the answer categories “gaining insight into the quality of care” was mentioned most often, followed by “participation was a condition for obtaining a contract” and “financial benefits” (see Table 2).

### Bivariate analysis

From 2009 to 2011, the percentage of participating physical therapists who only mentioned professional reasons to participate in Qualiphy decreased whereas the percentage of therapists that only mentioned financial reasons to participate increased (see Table 2). Chi squared tests proved these trends to be significant (χ^2^ = 7.47 respectively 6.73; p<0.05) (see Table 3). This is in line with the expectation of our first hypothesis and we can thus confirm that the reasons to participate varied when comparing the years of survey. Professional reasons were losing importance and financial reasons were gaining importance. Further, when we examine the bivariate association between the reasons to participate and the use of the feedback reports, we found that mentioning *only professional reasons* significantly correlates positively with using the feedback reports (Phi = 0.15; p<0.05) and that having *only financial reasons* correlates significantly negative with using the feedback reports (Phi = -0.27; p<0.001)(see Table 4). This is in line with hypothesis 2. Having a mixture of reasons to participate did not correlate significantly with the use of feedback reports. Last, the negative trend in using the feedback reports from 2009 to 2010 and 2011 as was seen in Table 2 (from 25.6% in 2009 who did not intend to use the reports to 44.1% in 2011) a Chi squared test found this trend to be significant (χ^2^ = 13.24; p<0.001)(see Table 3). In 2011, feedback reports were used significantly less than in the years before that, thus confirming hypothesis 3. These statistically significant bivariate relations were sufficient to proceed to logistic regression analysis.

**Table 3:** Chi squared test between the *reasons to participate* and the *year of survey*, and between *use of feedback reports* and *year of survey*.

	Only professional reasons	Only financial reasons	Mixture of reasons	Use of feedback reports
	χ^2^	χ^2^	χ^2^	χ^2^
Year of survey	7.47*	6.73*	6.80*	13.24***

*p<0.05,

**p<0.01,

***p<0.001.

**Table 4:** Phi correlation between *reasons to participate* and *the use of feedback reports*.

	Only professional reasons	Only financial reasons	Mixture of reasons
Use of feedback reports	0.15*	-0.27***	0.81

*p<0.05,

**p<0.01,

***p<0.001.

### Logistic regression analysis

Table 5 summarizes the results of the regression models. PTs that only mention professional reasons to participate have a higher odds to use the feedback reports than PTs with a mixture of reasons. This effect however is not significant (OR = 2.55; 95%CI = 0.93–6.82) (Model 1). However, PTs that only had financial reasons to participate have significantly lower odds than PTs with a mixture as reasons to use the feedback reports. This effect is significant (OR = 0.24;95%CI = 0.11–0.52). Despite the significant positive bivariate relation between only mentioning professional reasons to participate and the use of feedback reports, this relation did not hold when compared to PTs with a mixture of reasons. The PTs that only mentioned financial reasons to participate were less likely to use the feedback reports than PTs with mixed reasons, thus confirming hypothesis 2. Adding year of survey into the analysis (Model 2) did not change the effects or significance of the reasons to participate. PTs both in 2009 and 2010 had a significantly higher odds (p<0.01) than PTs in 2011 (reference category) to use the feedback reports (OR = 2.41;95%CI = 1.25–4.64 respectively OR = 3.28;95%CI = 1.51–7.10), thus confirming hypothesis 3. In Model 3 (not in Table 5), the control variables *age* and *gender* did not have significant effects, nor did they change the effects of Model 2. They were left out of the model estimation to keep the model as parsimonious as possible. Last, the model improved significantly from the constant-only model (not in Table 5) to Model 1 and Model 2 (Step χ^2^ = 20.39 respectively 12.06; p<0.01). Compared to the constant-only model, model 2 improved significant as well (Model χ^2^ = 32.45; p<0.001).

**Table 5:** Logistic regression analysis on the use of feedback reports.

	Model 1			Model 2		
	Exp(B)	95%CI	Wald’s Χ^2^	Exp(B)	95%CI	Wald’s Χ^2^
Reasons to participate:						
Mixture of reasons (Ref.)						
Only professional reasons	2.52	0.93–6.82	3.31	2.00	0.72–5.53	1.78
Only financial reasons	0.24***	0.11–0.52	13.16	0.21***	0.09–0.47	14.30
Year of survey:						
2009				2.41**	1.25–4.64	6.91
2010				3.28**	1.51–7.10	9.02
2011 (ref.)						
Constant	2.54			1.48		
Model Χ^2^ (df)		20.39(2)***			32.45(4)***	
Step Χ^2^ (df)		20.39(2)***			12.06(2)*	
N		257			257	

* p<0.05,

** p<0.01,

*** p<0.001.

### Qualitative data

The qualitative interviews show comparable participation aims as the quantitative data. Most of the interviewees mentioned a mixture of reasons to participate in the Qualiphy project. “Getting better contracts,” “obligated by the insurers” and “gaining insight in their own quality” were mentioned most often. As physiotherapist 4 mentioned:

"It was a bit imposed by the health insurers. The choice was between less payment per consultation or participate in Qualiphy and getting a better payment … that provokes joining the program. But it's not just about the financial incentives, it's also about the quality you offer patients. Both are important.”

The association between the reasons to participate and the use of the feedback reports is confirmed by the qualitative interviews. Three of PTs only mentioned the financial incentives to participate, none of whom used the feedback reports, although trust issues seem to be more of a driver not to use the feedback reports. As physiotherapist 1 mentioned:

“We have discussed it during the team meeting. As the general opinion in our practice was that the data was not reliable, we did not use the results.”

Most of the other PTs used the feedback reports at least to some extent. As physiotherapist 7 mentioned:

“The feedback report is clear and well-organized and complete, it takes relatively little time to go through and one can see which reality underlies the scores and what issues play. We have pretty quickly found possible improvements and have put action thereon.”

PTs who showed a mixture of reasons to participate, but did not use the feedback reports to improve their quality of care, reasoned that there was not much to improve or questioned the validity of the data. As physiotherapist 10 mentions:

"We scored very high in the domain physical therapy process and we did not expect anything else. However, the questionnaire on patient experiences last year was not appropriate for our patients, so I felt that it was more an indication than that we could really use the results, however, also these scores were quite high. We are just already very engaged in quality improvement."

### Barriers and facilitators

Barriers and facilitators were categorized in three subthemes, i.e. the feasibility of Qualiphy, the usability of the feedback reports, and policy issues.

A strong point of the Qualiphy project was that it served as a mirror or confirmation tool for reflection, it complemented already existing quality initiatives, and it helped to structure and to implement the guidelines for methodical reporting.

Physiotherapist 9: *“And in terms of quality*, *our reporting in the electronic health records has actually improved*.*”*

Physiotherapist 12: *"In our year planning the goals we want to achieve were already present*, *as well as improvement areas coming from the feedback reports*. *With the results of the feedback reports*, *you can make a better statement on quality improvement within your own practice*.*"*

Another facilitator was the possibility to zoom into the domain practice organization and patient experiences, and learn item-wise about the different aspects.

Physiotherapist 12: *"We have also looked at the results at item level*, *and that was really helpful*. *The results of the domain practice organization were really concrete*, *for example*, *whether the toilet was clean*. *This was really useful for us*.*”*

The direct extraction of the data from the Electronic Health Record (EHR) in 2011 was seen as a positive development both with respect to feasibility as to the reliability of the data. It was also mentioned often as a suggestion to improve the quality system. Data collection on the domain physical therapy process was judged as a barrier in 2009 and 2010 as the self-rating was qualified as sensitive to fraud. This affected the perceived reliability and thus the perceived usability of the feedback reports. Also, the validity of the data is questioned.

Physiotherapist 1: *"I did not like the study in 2009 and 2010*, *because all the therapists had to fill out all the questionnaires online*, *and there was no check if you filled out the survey honestly*. *In addition*, *with respect to the patient experiences*, *I think we all know that the physiotherapists have completed those themselves*. *Ultimately*, *I've done very little with the results because I thought it was not a clear reflection of reality*.*"*

Other barriers to acceptability were that the set of indicators was too generic and not applicable to (specialized) practices. This issue is also addressed when asked for suggestions to improve the set of quality indicators or feedback reports. More than half of the interviewees suggested that the set of indicators should be condition specific to do more justice to the different needs of and expectations for different conditions.

Furthermore, in the sub-theme policy, concerns for the role of the other stakeholders could also influence the attitude towards the quality system. The concerns were mainly directed at the professional body of PTs (KNGF) and the insurers. There is distrust towards both; the reputation of the KNGF is that they do not sufficiently represent the interest of the PTs, but rather want to be of service to the insurers. The role of the insurers is also questioned because they have changed the rules during the implementation process. The initial goal of the insurers was to use the tool to reward quality, but it turned into a punishing tool for PTs who did not want to participate, or did not include enough patients.

Following from above a general feeling of unease, be it with respect to the quality of the data or to the role of the stakeholders, can have a negative effect on the use of the quality instrument for quality improvements. For physical therapy practices that already had quality of care high on the agenda, the feedback reports help to support and structure those efforts.

## Discussion

Our study showed that the percentage of PTs that only had professional reasons to participate decreased from 2009 to 2011 and at the same time the PTs that only mentioned financial reasons to participate increased from 2009 to 2011. The increase in the percentage of PTs that only showed interest in the obtaining financial benefits, or evading financial punishment had a negative effect on the use of the feedback reports for quality improvements. Controlled for the negative effect of only having financial reasons to participate on the use of feedback reports, PTs in 2009 and 2010 were more likely to use the feedback reports for quality improvement than the PTs in 2011. During the project, the circumstances surrounding the Qualiphy project changed, for example with respect to the perceived voluntariness to participate linked to financial rewards and penalties, as well as retrieving data directly from EHRs instead of by self-reporting. From the qualitative interviews, a picture of growing distrust emerges, both with respect to the reliability of the data and the quality indicators, as well as between participating PTs and health insurance companies. This was associated with a decrease in the willingness to use the feedback reports and can be seen as the most important barrier for a successful implementation of quality improvement strategies. Our findings were confirmed by the quantitative as well as the qualitative data. The most important facilitator for using the feedback reports is that is can serve as a tool to support and structure quality improvement initiatives. The mixed methods approach in this study can be considered an added value as it deepened our understanding of the results.

This paper finds clear evidence that financial incentives can have an adverse effect on the desired behavior, that is quality improvements, although three reviews on the effects of external incentives on the desired behavior (be it using the guidelines, or working evidence-based) found the evidence to be inconclusive [11–13]. This paper contributes to the scientific knowledge on the use of financial incentives in quality improvement initiatives.

This is not the complete story however. In 2011, the percentage of PTs that only participated for financial reasons was higher than it was in 2009, which was most likely caused by different consequences of participating in the different cohorts affecting the group of participants. In 2009, participation was voluntary, so the PTs that joined the project at that time were the most willing to learn from the project and to change, i.e. to improve the quality of care. In 2010, participation was rewarded and in 2011 it was mandatory to obtain a contract. The groups that joined in 2010 and 2011 may be seen as more reluctant to take part in this project, but felt that they did not have a choice. So the group of PTs that had professional reasons to participate may have become relatively small and the effects on the use of feedback reports might have been a composition effect due to a relatively large group of PTs in 2010 and 2011 who felt a ‘force’ to participate due to payment repercussions.

The data further showed that in 2011, it was less likely that the feedback reports were used compared to 2009, apart from the influence of motivation. The incentives were aimed at stimulating participation and at ensuring that the required number of patients were included instead of stimulating the use of feedback reports for quality improvements, for example, by yielding a higher reward for high quality performance. Especially for the PTs who were not eager to participate in the project to begin with, the incentives were misdirected. The mid-game change of rules and the introduction of external control mechanisms led to distrust among the participating PTs towards health insurers. There already was distrust towards the reliability of the audit and feedback system from the beginning of the project, as the 2009 and 2010 data collection was by means of self-reporting. PTs felt that this method was prone to gaming and thus the results were not representative of reality. The introduction of external control mechanisms in 2010 and 2011 (only obtaining contract if you participate in the project) and the change in data collections methods might have added to distrusting the outcomes of the project and thus to a smaller chance that feedback reports were taken seriously. This supports the substitution perspective of the relationship between trust and control [25,26]. In this perspective, trust and (formal) control are inversely related, so when control increases, trust decreases. Trust and control cán be mutually reinforcing according to the complementary perspective [26] in which control mechanisms can help to build trust if people are provided with objective rules and clear measures. Greezny et al. [16] also conclude that incentives can have (moderate) effects on behavior when they are well specified and well targeted. The change in rules during Qualiphy was neither objective nor clear and the incentives were not directed at quality improvements.

In the end, the feedback reports were being used less and quality improvements as a result of the Qualiphy project decreased. Deci and Ryan [14,15] predicted this adverse behavior in their studies. In the Cognitive Evaluation Theory, a sub theory of the Self-Determination Theory, they describe that to maintain a high level of intrinsic motivation, a subject has to have a sense of autonomy in their behavior [14,15]. With the introduction of financial rewards and punishments, that sense of autonomy is affected. A subgroup analysis of the PTs that participated voluntarily in the first wave and participated in the two following waves as well seems to support that idea. When this group is analyzed, the same trend from professional reasons to financial reasons can be seen and a smaller likelihood to use the feedback reports (data not shown). However, these subgroups are too small to draw scientifically sound conclusions from.

### Implications for practice and research

Health insurers and professional organizations should be aware of the influence of the reasons to participate in quality improvement initiatives on the result and the effectiveness of the quality systems. Imposing financial incentives (both rewards and punishments) is only effective when therapists are already interested, believe in the instrument itself, when rules are clear and the incentives are directed at either improving the quality of care, or reaching a certain pre-set level of quality or are related to the necessary time investment of data sampling. In such conditions financial incentives can strengthen intrinsic motivation, but in the Qualiphy project a pay-for-participation scheme was used, not connected to actual performance. A number of participating practices had a drop in income as a result of received penalties because they did not include enough patients. Future quality systems should emphasize tailored rewarding rather than punishing in addition to creating and stimulating the motivation of professional nature, and an investment should be made to develop and maintain a trustworthy relationship between insurers and PTs. To develop such a system takes time and should not be rushed. There is some evidence that it is better to develop the system bottom-up with small groups of professionals, for example in quality circles [27] in which small groups of professionals meet at regular intervals, to discuss their individual feedback reports with benchmarks. Such groups should aim at assessing and improving the quality of care and work autonomously. These assumptions are supported by evidence that step-by-step quality improvements are possible [27–30].

Some of the PTs that were interviewed showed classic defense mechanisms [31,32] when the outcomes of the feedback reports were not as expected, that is to reject the results completely and claim the data was not reliable, or the survey questions were not suitable for their exceptional situation or deviant patient population, as was shown from the qualitative data. This again shows the importance of building trust among users of quality measuring projects.

### Future research

Future research should elaborate on the findings of this study, as it is the first to link the reasons to participate in quality systems with the use of feedback reports. More research is needed however to better understand the mechanisms and find potential confounders of the studied effects. Friedberg et al. [33] found for example that the organizational structure, such as practice size and team climate had an influence on the use of patient experiences data for quality improvements. Taking practice size into account can potentially explain some of the variation in the use of feedback reports. Larger practices have more resources to gather data and to design improvement plans based on the feedback reports. This was also mentioned in the interviews. A therapist from a small practice claimed she simply did not have the time to implement extensive quality improvement policies. They had enough trouble meeting the requirements of health insurers besides the actual treatment of their patients.

### Limitations

This study has some limitations. First, the subgroup of therapists that showed only financial reasons to participate in the Qualiphy project was relatively small. Caution as to the results of this subgroup is therefore necessary. However, the effects were highly significant and did not have a large confidence interval, which would be a signal for unreliable effects.

Second, the data that was used was part of a broader evaluation survey, not specifically directed at understanding the factors influencing the use of feedback reports. More data is necessary to help interpret our finding that in 2011 the use of feedback reports was negatively affected.

The samples of the evaluation surveys were rather small, so sub-group analysis was not always possible. Further, it could decrease the generalizability of the study, as no non-response analysis was performed. It could be possible that PTs that were positive about the feedback reports were the ones willing to fill out the evaluation survey, overestimating the use of the feedback reports. However, earlier research on the quality indicators showed large ceiling effects and low variance in indicator scores, so the chance of sampling PTs with positive feedback reports was quite high [20]. We therefore feel it is justified to generalize the finding to the whole population of participating PTs.

A last limitation is that the use of feedback reports is measured by self-reporting. This might have induced more positive responses overestimating the real use of the feedback reports. Also, as was mentioned in the interviews with the PTs, not using the feedback reports could have been induced by a high score on the indicators. If the measured quality already was very high, this would reduce the need for changes.

## Conclusion

Having only financial reasons to participate in quality systems has the strongest negative influence on the use of feedback reports to improve the quality of care. Policy makers should be aware that introducing external stimuli mid-game when implementing a quality system can decrease the trust necessary to improve the quality of care. If clinicians do not believe in the quality system, they will be less likely to use the system to their benefit and to the benefit of the patients. In the end, the main goal of quality systems is to ensure patients of the best care possible.
